# Structural and Functional Alterations of MAMs and Their Immunomodulatory Roles in Sepsis‐Induced Lung Injury

**DOI:** 10.1155/jimr/9888339

**Published:** 2026-04-30

**Authors:** Yihao Wang, Jingran Yang, Xia Li

**Affiliations:** ^1^ Department of Thoracic Surgery, Affiliated Hospital 6 of Nantong University, Yancheng Third People’s Hospital, Yancheng, 224000, China, jsycsy.com; ^2^ Medical School of Nantong University, Nantong, 226007, China, ntu.edu.cn; ^3^ First Clinical Medical College, Xuzhou Medical University, Xuzhou, 221004, Jiangsu, China, xzmc.edu.cn; ^4^ Department of General Medicine, Affiliated Hospital 6 of Nantong University, Yancheng Third People’s Hospital, Yancheng, 224000, China, jsycsy.com

**Keywords:** endoplasmic reticulum, immune regulation, inflammatory response, MAMs, mitochondria, septic lung injury, structural and functional changes

## Abstract

**Background:**

Sepsis‐induced acute lung injury (SI‐ALI) is a major cause of morbidity and mortality among septic patients. Recent evidence highlights the role of mitochondria‐associated membranes (MAMs)—specialized contact sites between the endoplasmic reticulum (ER) and mitochondria—in regulating calcium signaling, lipid metabolism, energy homeostasis, and immune responses. Structural and functional alterations of MAMs are increasingly recognized as critical contributors to the pathogenesis of SI‐ALI.

**Objectives:**

This review aims to summarize the structural and functional characteristics of MAMs, elucidate their alterations and immunoregulatory roles in sepsis‐induced lung injury, and discuss potential therapeutic strategies targeting MAMs to mitigate pulmonary damage.

**Methods:**

A comprehensive literature review was conducted using recent studies focused on the molecular structure, signaling mechanisms, and pathological changes of MAMs in sepsis and related inflammatory diseases. Emphasis was placed on calcium signaling, mitochondrial dysfunction, oxidative stress, and inflammasome activation.

**Results:**

MAMs maintain close ER–mitochondria contacts (10–30 nm) through key proteins such as inositol 1,4,5‐trisphosphate receptor (IP3R), glucose‐regulated protein 75 (GRP75), voltage‐dependent anion channel (VDAC), and mitofusin‐2 (MFN2). During sepsis, oxidative stress and inflammatory cytokines disrupt these contacts, leading to impaired calcium transfer, mitochondrial dysfunction, and energy deficiency. Dysregulated MAMs promote NLR family pyrin domain containing 3 (NLRP3) inflammasome activation, excessive reactive oxygen species (ROS) production, and mitochondrial DNA (mtDNA) release, thereby amplifying inflammatory cascades and immune cell apoptosis. Therapeutic strategies that restore MAM integrity—such as upregulating MFN2, activating ER autophagy, or modulating calcium transport proteins—have shown potential to attenuate lung injury by improving mitochondrial metabolism and reducing oxidative stress.

**Conclusions:**

MAMs play essential roles in maintaining intracellular homeostasis and immune balance. Their structural and functional disruption contributes significantly to the progression of SI‐ALI. Targeting MAMs offers promising therapeutic opportunities for preventing and treating sepsis‐induced lung injury, although further mechanistic and clinical studies are warranted to translate these findings into practice.

## 1. Introduction

Sepsis‐induced lung injury is one of the most severe complications in patients with sepsis and has become a leading cause of mortality. The onset of sepsis is typically accompanied by systemic inflammatory response syndrome (SIRS), a complex pathological process involving multiple interacting mechanisms. Uncontrolled inflammation is a hallmark of sepsis, during which immune cells become excessively activated under infectious stimuli, releasing large amounts of proinflammatory mediators that contribute to lung tissue injury. Organelle dysfunction—particularly involving the endoplasmic reticulum (ER) and mitochondria—also plays a critical role in the pathogenesis of sepsis‐induced lung injury. Disruption of intracellular energy metabolism and calcium homeostasis further exacerbates lung tissue damage and cell death.

In recent years, increasing attention has been paid to the interactions between the ER and mitochondria. The mitochondria‐associated membranes (MAMs), which serve as key contact sites between these two organelles, act as crucial platforms regulating various physiological and pathological cellular processes. MAMs are essential for maintaining intracellular calcium homeostasis, regulating energy metabolism, and mediating signal transduction. Studies have shown that the structure and function of MAMs undergo significant alterations in various diseases, potentially affecting inflammatory responses, apoptosis, and cell survival. In the context of sepsis‐induced lung injury, such alterations in MAMs may profoundly influence pulmonary inflammation and cell fate.

Current research indicates that MAMs exhibit marked structural and functional changes during sepsis‐induced lung injury. These alterations disrupt the intracellular calcium balance, impair mitochondrial energy metabolism, and lead to energy deficiency, thereby promoting apoptosis and necrosis. Moreover, abnormal MAMs may facilitate the release of inflammatory cytokines, further aggravating pulmonary inflammation. Understanding these mechanisms provides new insights into the pathophysiology of sepsis‐induced lung injury and opens potential avenues for therapeutic intervention.

This paper aims to systematically summarize the structural and functional alterations of MAMs and their immunoregulatory roles in sepsis‐induced lung injury. It further explores the mechanisms by which MAMs contribute to the development of this condition and discusses their potential clinical significance. Through an in‐depth analysis of MAM‐related functional changes, we hope to provide novel perspectives for the prevention and treatment of sepsis‐induced lung injury, ultimately improving patient outcomes and reducing sepsis‐associated mortality.

## 2. Methods

This narrative review was based on a comprehensive literature search of the PubMed, Web of Science, and Scopus databases. Relevant articles were identified using combinations of keywords including “mitochondria‐associated membranes” or “MAMs,” “sepsis,” “acute lung injury,” “ARDS,” “calcium signaling,” “mitochondrial dysfunction,” “inflammasome,” and “oxidative stress.”

Original research articles and review papers published in English were considered. Studies focusing on the structure and function of MAMs, their role in inflammatory signaling, mitochondrial dysfunction, immune regulation, and lung injury in the context of sepsis were preferentially included. Papers unrelated to sepsis, lung injury, or MAM‐associated mechanisms were excluded.

The literature search primarily covered publications from the past 10–15 years, with particular emphasis on recent studies that provide mechanistic insights into MAM dysregulation and its therapeutic implications in sepsis‐induced lung injury. Additional relevant articles were identified through manual screening of reference lists.

## 3. Structural and Functional Basis of MAMs

### 3.1. Structural and Functional Basis of MAMs

#### 3.1.1. Composition and Structural Characteristics of MAMs

**Figure 1:**
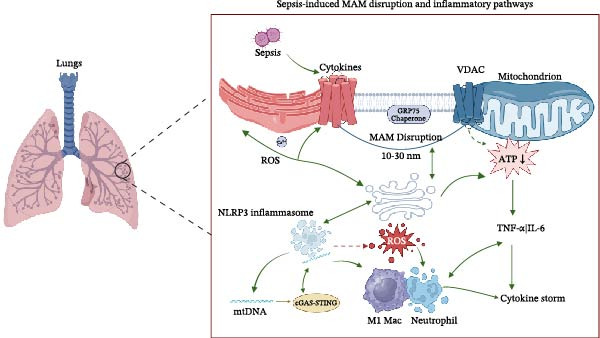
This figure provides a conceptual overview of sepsis‐induced lung injury, emphasizing mitochondria‐associated membranes (MAMs) as a signaling hub. Sepsis‐associated stimuli disrupt MAM integrity, leading to Ca^2+^ dysregulation and excessive reactive oxygen species (ROS) production. These alterations are associated with inflammasome activation and increased release of proinflammatory cytokines (e.g., TNF‐α and IL‐6), contributing to immune dysfunction and an amplified inflammatory response. Key MAM‐related components (IP3R, GRP75, VDAC, and MFN2) are highlighted to illustrate their roles in ER–mitochondria communication and downstream inflammatory consequences.

Mitochondria‐associated endoplasmic reticulum membranes are specialized contact sites between the ER membrane and the outer mitochondrial membrane, typically separated by ~10–30 nm [1]. This close association supports intracellular signaling and metabolite exchange. Core MAM components include the inositol 1,4,5‐trisphosphate receptor (IP3R), the voltage‐dependent anion channel (VDAC), glucose‐regulated protein 75 (GRP75), and mitofusin‐2 (MFN2), which collectively mediate ER–mitochondria tethering and interorganelle signal transduction [2, 3]. A comprehensive summary of these MAM‐associated proteins and their functional roles in sepsis‐induced lung injury is provided in Table 1. The organization of the core ER–mitochondria tethering and Ca^2+^‐transfer complex at MAMs is schematically illustrated in Figure 1.

**Table 1:** Key MAM‐resident proteins and their roles in the pathogenesis of sepsis and ALI.

Protein (gene)	MAM localization/complex	Primary function at MAMs	Change in sepsis/ALI	Downstream consequence	Therapeutic note/relevance
IP3R	ER, Ca^2+^ release axis	ER Ca^2+^ release to mitochondria	Downregulated/dysregulated [4, 5]	Ca^2+^ transfer impaired → mitochondrial dysfunction, ATP↓, cell death↑	Potential target to normalize Ca^2+^ flux
GRP75	Bridge (IP3R–VDAC)	Chaperone linking IP3R and VDAC	Oxidative modification/dysregulated [6]	Ca^2+^ signaling inefficiency; stress amplification	MAM stabilization strategy
VDAC	OMM, Ca^2+^ entry axis	Mitochondrial outer membrane Ca^2+^ conduit	Downregulated/dysregulated [4, 5]	Mitochondrial Ca^2+^ handling abnormal → ROS↑/apoptosis↑	Candidate for Ca^2+^‐modulating therapy
MFN2	Tethering/architecture	ER–mitochondria tethering, MAM integrity	Reduced expression [7]	Contact area↓/distance↑ → Ca^2+^ homeostasis disrupted; bioenergetics impaired	TMP upregulates MFN2 and promotes MAM recovery [8]
Sig1R	ER/MAM modulator	Stress signaling modulation, MAM function support	Therapeutic activation beneficial [9]	Inflammation attenuated; endothelial protection	Sig1R activation ameliorates sepsis‐induced lung injury [9]
FAM134B	ER‐phagy related	ER‐phagy regulation; affects MAM remodeling	Regulated in ALI model [10]	ER stress/autophagy balance → excessive MAM formation reduced	Dendrobine via FAM134B improves lung injury [10]

MAM structural stability is essential for intracellular Ca^2+^ signaling and lipid transport, thereby supporting metabolic homeostasis. MAM function is regulated by the expression and interaction of its constituent proteins, and alterations in these components can affect MAM formation and stability. For example, the IP3R–VDAC interaction facilitates ER‐to‐mitochondria Ca^2+^ transfer, while GRP75 bridges these proteins to promote efficient signal transmission [11, 12].

MAM architecture is dynamically modulated under physiological and pathological conditions. Beyond Ca^2+^ homeostasis, MAMs also serve as platforms for lipid metabolism, mitochondrial stress responses, and autophagy, highlighting their multifaceted role in coordinating cellular physiology [13].

The major MAM‐associated proteins, their core functions, reported changes in sepsis/acute lung injury, and potential therapeutic relevance are summarized in Table 1.

#### 3.1.2. Physiological Functions of MAMs

In cellular physiology, mitochondria‐associated endoplasmic reticulum membranes function as critical structural domains that play indispensable roles in maintaining cellular homeostasis. Beyond serving as physical bridges between mitochondria and the ER, MAMs are deeply involved in a wide range of physiological processes. One of their most essential functions lies in the regulation of intracellular calcium homeostasis. Through interactions with IP3Rs located on the ER, MAMs facilitate the transfer of calcium ions from the ER to mitochondria—a process crucial for maintaining calcium equilibrium and modulating cellular physiological activities [14, 15].

MAMs also participate actively in lipid metabolism, particularly in the synthesis and trafficking of phospholipids and cholesterol. Owing to their unique structural organization, MAMs serve as hubs for lipid exchange and metabolic regulation, contributing to membrane biogenesis and interactions with other organelles. Dysregulation of MAMs has been closely linked to the pathogenesis of various diseases, including cardiovascular disorders and metabolic syndromes [16, 17].

Furthermore, MAMs play vital roles in cellular energy metabolism and apoptotic signaling. By modulating mitochondrial dynamics and bioenergetic functions, MAMs help sustain cellular energy balance. Under stress or apoptotic stimuli, alterations in MAM function can influence the decision between cell survival and death. Evidence suggests that MAMs regulate apoptosis by controlling calcium signaling and participating in autophagic processes, thereby exerting profound effects on cell fate determination [18, 19].

Overall, MAMs act as multifunctional regulators in cellular physiology, and the integrity of their structure and function is fundamental to cellular health. Comprehensive investigations into the roles of MAMs not only enhance our understanding of their physiological importance but also provide new perspectives for exploring their pathological alterations. As research on MAMs continues to advance, novel therapeutic targets may emerge, offering promising avenues for the treatment of MAM‐related diseases [20, 21].

#### 3.1.3. Roles of MAMs in Immune Signaling

Mitochondria‐associated endoplasmic reticulum membranes serve as critical intracellular signaling hubs that play a pivotal role in regulating immune responses. By facilitating the physical and functional interplay between the ER and mitochondria, MAMs enhance the cellular response to inflammatory stimuli. During acute inflammation, MAMs modulate calcium (Ca^2+^) transport and lipid metabolism to promote the activation of the NLR family pyrin domain containing 3 (NLRP3) inflammasome, subsequently triggering the release of proinflammatory cytokines [22].

The NLRP3 inflammasome is a key multiprotein complex in innate immunity, and its activation is closely associated with the pathogenesis of numerous inflammatory diseases. Upon infection or cellular injury, MAMs contribute to NLRP3 assembly and activation by regulating ER stress and mitochondrial function. This process results in the release of proinflammatory cytokines such as interleukin‐1β (IL‐1β), a mechanism that has been substantiated across multiple disease models [23, 24].

In addition, MAMs influence NLRP3 inflammasome activation through regulation of the intracellular redox state. Under oxidative stress conditions, the structural and functional integrity of MAMs may become compromised, leading to aberrant NLRP3 activation. Such dysregulation has been particularly evident in metabolic disorders, including diabetes and cardiovascular diseases, where impaired MAM function appears to exacerbate disease progression [25, 26].

**Figure 2:**
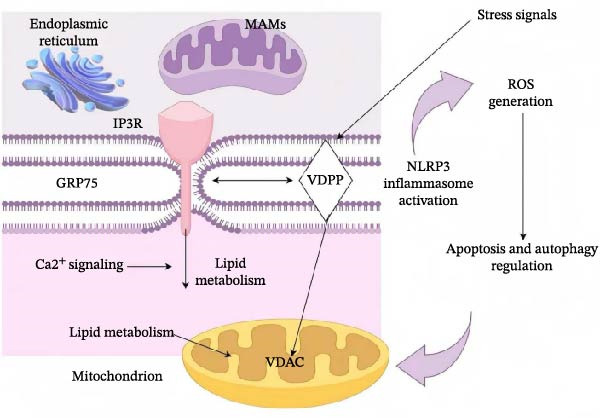
The figure depicts the structural and functional relationship between the endoplasmic reticulum (ER) and mitochondria at the mitochondria‐associated membranes (MAMs). Key tethering proteins—including inositol 1,4,5‐trisphosphate receptor (IP3R), glucose‐regulated protein 75 (GRP75), and voltage‐dependent anion channel (VDAC)—mediate calcium (Ca^2+^) transfer and lipid metabolism between the two organelles. MAMs serve as critical hubs for regulating Ca^2+^ signaling, lipid metabolism, and mitochondrial function. Under stress conditions, excessive reactive oxygen species (ROS) are generated, leading to the activation of the NLRP3 inflammasome and triggering apoptotic and autophagic responses. This integrative network highlights the pivotal role of MAMs in coordinating metabolic and inflammatory signaling pathways in cellular homeostasis and stress adaptation.

Taken together, MAMs act not only as integrative platforms for inflammatory signaling but also as key modulators of immune responses. Further elucidation of their roles in immune regulation will be essential for understanding the mechanisms underlying inflammation and may provide novel therapeutic targets for the treatment of inflammation‐related diseases. These findings highlight that MAMs function as critical signaling hubs integrating calcium transfer, lipid metabolism, and inflammatory responses. A schematic overview of MAM‐mediated signaling pathways is illustrated in Figure 2, summarizing their structural organization and immunoregulatory mechanisms.

### 3.2. Structural Alterations of MAMs in Sepsis‐Induced Lung Injury

#### 3.2.1. Disruption of MAM Structural Integrity

Under septic conditions, oxidative stress leads to the excessive production of reactive oxygen species (ROS), which not only directly damage cellular components but also induce oxidative modifications of key MAM‐associated proteins. These modifications can result in downregulation of their expression or impairment of their function, thereby disrupting MAM integrity [6, 23]. In addition, inflammatory cytokines such as tumor necrosis factor (TNF) and interleukin‐6 (IL‐6) are markedly elevated during sepsis. These cytokines can further suppress MAM function through multiple signaling pathways, consequently affecting its roles in cellular metabolism and signal transduction. IL‐6, in particular, has been shown to promote ER stress, thereby compromising the structural and functional integrity of MAMs and leading to disturbances in intracellular calcium homeostasis [27].

In sepsis, the expression of key calcium‐signaling proteins within MAMs—such as IP3R and VDAC—is often downregulated. This dysregulation exacerbates cellular dysfunction by impairing calcium transfer and disrupting metabolic and survival pathways [4, 5]. Pathologically, sepsis is characterized by a reduction in the contact area between mitochondria and the ER, as well as abnormalities in the intermembrane distance. Under physiological conditions, MAMs maintain an optimal spacing of ~10–30 nm, which is critical for efficient signal transmission. A decrease in contact area or an increase in distance between these membranes can lead to diminished calcium signaling efficiency, thereby impairing the cell’s ability to respond to external stimuli [12, 28].

The integrity of MAMs is essential for effective calcium transfer, and structural disruption of MAMs markedly reduces calcium signaling efficiency. This impairment in turn compromises mitochondrial energy metabolism and overall cell viability. Alterations in MAM architecture have been closely associated with multiple organ dysfunction in sepsis. As a consequence of MAM abnormalities, mitochondrial function becomes inhibited, leading to insufficient energy production and enhanced apoptosis—pathophysiological features commonly observed in septic patients [29, 30].

#### 3.2.2. Alterations in Membrane Protein Expression and Posttranslational Modifications

Under septic conditions, membrane‐associated proteins are highly susceptible to phosphorylation, a posttranslational modification that can alter protein stability and interactions with other molecules, thereby influencing cellular inflammatory responses and overall function [2]. During sepsis, the expression of membrane proteins such as MFN2 is significantly reduced, leading to weakened membrane tethering and impaired intracellular calcium signaling. MFN2, a key protein mediating the physical connection between mitochondria and the ER, plays an essential role in maintaining interorganelle communication. Its downregulation disrupts this interaction, resulting in disturbances in calcium homeostasis and cellular metabolic processes [7].

Ubiquitination of membrane proteins also plays a crucial role in the pathophysiology of sepsis. While ubiquitination is traditionally recognized as a marker for protein degradation, it can also modulate protein function and molecular interactions. Under septic conditions, the elevated ubiquitination levels of certain membrane proteins may suppress or alter their activity, thereby contributing to cellular stress responses and inflammatory signaling [31].

In this context, investigating the posttranslational modifications of membrane proteins provides valuable insights into the molecular mechanisms underlying sepsis‐induced cellular dysfunction. The impact of altered membrane protein expression and modification during sepsis extends beyond inflammation, encompassing the regulation of cell survival and death. Changes in membrane protein profiles can influence intracellular signaling pathways, thereby triggering biological processes such as apoptosis and autophagy, which are central to the progression of sepsis‐induced cellular injury [32].

#### 3.2.3. Functional Consequences of Structural Alterations in MAMs

Structural alterations in mitochondria‐associated endoplasmic reticulum membranes exert profound effects on cellular function, particularly in diseases such as sepsis‐induced lung injury. Disruption of MAM integrity can lead to impaired calcium ion (Ca^2+^) transfer between the ER and mitochondria, resulting in widespread disturbances in cellular homeostasis. The structural breakdown of MAMs compromises intracellular calcium regulation and causes mitochondrial calcium overload. Calcium plays a pivotal role in cellular signal transduction, especially in modulating mitochondrial function and apoptosis. The structural integrity of MAMs is therefore essential for efficient calcium transport; when calcium transfer from the ER to mitochondria is impaired, mitochondrial adenosine triphosphate (ATP) production decreases, leading to metabolic dysfunction and reduced cell viability [27].

Abnormal calcium flux also activates multiple proinflammatory signaling pathways, amplifying cellular inflammatory responses. This heightened inflammation not only exacerbates cellular injury but also accelerates apoptosis. Moreover, MAM damage contributes to the dysregulation of cellular energy metabolism, aggravating lung tissue injury. As a critical communication interface between mitochondria and the ER, the integrity of MAMs is vital for maintaining mitochondrial function and energy metabolism. Structural disruption of MAMs can impair mitochondrial oxidative phosphorylation and ATP synthesis, leading to cellular energy deficiency and functional decline [21]. In the context of sepsis‐induced lung injury, such metabolic disturbances hinder tissue repair and regeneration while promoting apoptosis of alveolar epithelial cells, thereby worsening pulmonary damage.

Furthermore, structural changes in MAMs may influence autophagy and apoptosis. Studies have demonstrated that MAM dysfunction is closely associated with autophagy inhibition—a process essential for maintaining intracellular homeostasis and removing damaged cellular components. Under pathological conditions such as sepsis, MAM impairment can compromise autophagic activity, thereby promoting apoptosis and exacerbating cell death and tissue injury [33].

### 3.3. Functional Changes of MAMs and Their Mechanisms in Sepsis‐Induced Lung Injury

#### 3.3.1. Dysregulation of Calcium Signaling

Calcium signaling is central to cellular homeostasis and is critically involved in sepsis‐induced lung injury. As key ER–mitochondria contact sites, MAMs regulate intracellular Ca^2+^ transfer and help maintain mitochondrial membrane potential and function. When MAM structure and function are compromised, ER Ca^2+^ handling becomes dysregulated, which can lead to mitochondrial Ca^2+^ overload and subsequent mitochondrial dysfunction, ultimately promoting apoptosis or necrosis.

Ca^2+^ imbalance also disrupts mitochondrial energy metabolism and alters cytosolic Ca^2+^ levels, thereby affecting multiple downstream pathways. Elevated intracellular Ca^2+^ can activate Ca^2+^‐dependent enzymes (e.g., phospholipase A_2_ [PLA_2_] and protein kinase C [PKC]), which is associated with increased ROS production and oxidative stress, further aggravating cellular injury and inflammation.

In addition, aberrant Ca^2+^ signaling amplifies inflammatory responses in sepsis‐induced lung injury. Increased intracellular Ca^2+^ can activate transcription factors such as nuclear factor kappa B (NF‐κB), upregulating inflammation‐related genes and enhancing proinflammatory cytokine release. This escalation of inflammation can worsen lung tissue damage and contribute to a self‐amplifying cycle of injury and inflammation.

#### 3.3.2. Mitochondrial Dysfunction and Oxidative Stress

Mitochondria‐associated endoplasmic reticulum membranes provide a critical signaling interface between the mitochondria and the ER, participating in the regulation of intracellular calcium transport, lipid metabolism, and oxidative stress responses. When MAM function is impaired, mitochondrial respiratory chain efficiency declines markedly, resulting in reduced ATP synthesis and consequent energy deficiency, which disrupts normal cellular function. In sepsis models, attenuation of MAM activity is closely associated with dysregulated cellular energy metabolism, thereby exacerbating pulmonary injury. Impaired mitochondrial calcium uptake leads to abnormal elevations in cytosolic calcium levels, which not only disturb normal intracellular signaling but also promote apoptosis and other pathological alterations [34].

During mitochondrial energy production, ROS are generated as natural byproducts of oxidative phosphorylation. Under physiological conditions, ROS serve as signaling molecules that modulate various cellular processes. However, under pathological conditions such as sepsis, mitochondrial dysfunction leads to excessive ROS production, triggering oxidative stress. This oxidative stress damages cellular structures and functions and simultaneously activates a cascade of inflammatory responses, forming a vicious cycle of injury and inflammation.

Excessive ROS further stimulate the release of proinflammatory cytokines, including TNF‐alpha (TNF‐α) and IL‐6, which amplify both local and systemic inflammatory responses. This escalation of inflammation contributes to progressive tissue damage, thereby worsening organ dysfunction and disease severity in sepsis‐induced lung injury [34, 35].

#### 3.3.3. Disruption of Lipid Metabolism

Under septic conditions, the function of mitochondria‐associated endoplasmic reticulum membranes is severely impaired, leading to disruptions in lipid transport. This impairment primarily affects the synthesis of phospholipids and cholesterol, resulting in compromised integrity of the cellular membrane. The structure and function of the cell membrane are fundamental to maintaining normal physiological activity, and membrane disruption can lead to cellular dysfunction and apoptosis, thereby exacerbating lung tissue injury. Impaired MAM‐mediated lipid transfer reduces intracellular phospholipid synthesis and alters the composition of membrane phospholipids, consequently affecting membrane fluidity and stability. Cholesterol, as a vital structural component of cellular membranes, also contributes to maintaining membrane physical properties; its decreased synthesis weakens the cell’s ability to resist external stress and insults.

In sepsis, damage to alveolar epithelial and endothelial cells compromises the pulmonary barrier function, promoting inflammatory cell infiltration and the release of inflammatory mediators. Abnormal lipid metabolism not only disrupts membrane integrity but also facilitates the production and secretion of proinflammatory mediators. Cytokines and chemokines released under these conditions amplify both local and systemic inflammatory responses, forming a vicious cycle that further aggravates lung injury.

Elevated levels of proinflammatory cytokines, such as TNF‐α and IL‐6, contribute to the accumulation of alveolar fluid and tissue edema, thereby impairing gas exchange and increasing the risk of respiratory failure. Moreover, disturbances in lipid metabolism can exacerbate lung injury indirectly by modulating immune cell function. Altered lipid profiles influence macrophage polarization, promoting a shift toward the proinflammatory M1 phenotype, which secretes higher levels of cytokines and further intensifies pulmonary inflammation.

#### 3.3.4. Potential Involvement of Ferroptosis in MAM Dysfunction–Associated Lung Injury

In addition to apoptosis and inflammasome‐mediated inflammation, ferroptosis has emerged as a distinct form of regulated cell death characterized by iron‐dependent lipid peroxidation and may also contribute to sepsis‐induced lung injury [36]. Oxidative stress and mitochondrial dysfunction, both prominent features of MAM dysregulation during sepsis, are recognized as critical drivers of ferroptotic cell death.

MAMs play an important role in lipid metabolism, redox homeostasis, and mitochondrial function. Disruption of MAM integrity may therefore create a permissive environment for lipid peroxidation by enhancing mitochondrial ROS production and altering membrane lipid composition, thereby potentially facilitating ferroptosis in pulmonary epithelial and endothelial cells. Although direct evidence linking MAM dysfunction to ferroptosis in sepsis‐induced lung injury remains limited, emerging studies suggest that ferroptosis may act in parallel with inflammasome activation and other forms of cell death to amplify tissue injury and inflammation.

Further investigation into the interplay between MAM dysregulation and ferroptosis may provide additional insights into the mechanisms of lung injury in sepsis and identify novel therapeutic opportunities.

### 3.4. Roles and Mechanisms of MAMs in Immune Regulation

#### 3.4.1. MAM‐Mediated Inflammasome Activation

Mitochondria‐associated endoplasmic reticulum membranes function as intracellular signaling hubs and contribute to multiple processes, including calcium transport, lipid metabolism, oxidative stress regulation, and apoptosis [37, 38]. Increasing evidence indicates that MAMs regulate the activation of the NLRP3 inflammasome, a multiprotein complex implicated in diverse pathological conditions, including diabetes, cardiovascular diseases, and sepsis [24, 39]. By providing a structural platform, MAMs can facilitate NLRP3 assembly and activation during cellular injury or infection, a process commonly associated with excessive ROS production and aberrant Ca^2+^ signaling [40].

Under septic conditions, MAM disruption can promote exaggerated NLRP3 activation and amplify inflammatory responses. In septic mouse models, impaired MAM integrity is associated with increased ROS production, enhanced NLRP3 inflammasome activation, and elevated release of IL‐1β and interleukin‐18 (IL‐18), thereby aggravating systemic inflammation and tissue injury [41, 42]. MAM dysfunction may also increase mitochondrial Ca^2+^ loading, intensifying oxidative stress and inflammation, which can contribute to disease severity and multiorgan dysfunction [38, 43].

**Figure 3:**
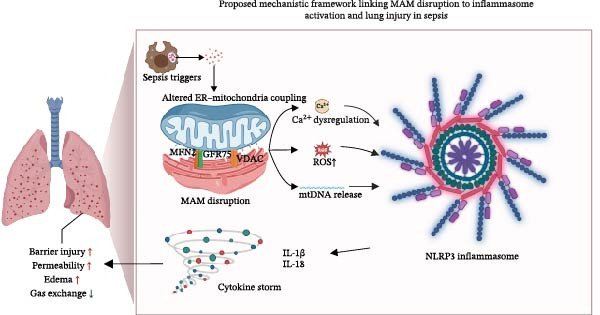
This figure illustrates how sepsis‐related triggers (PAMPs/DAMPs, proinflammatory cytokines, and hypoxia) perturb ER–mitochondria coupling at MAMs by affecting key tethering and signaling components (e.g., MFN2 and the IP3R–GRP75–VDAC axis). MAM dysfunction results in dysregulated Ca^2+^ transfer, increased mitochondrial ROS generation, and mitochondrial DNA (mtDNA) release. These mitochondrial danger signals converge on NLRP3 inflammasome activation, promoting IL‐1β and IL‐18 maturation and release and amplifying inflammatory cascades/cytokine storm. The resulting exaggerated inflammation contributes to pulmonary barrier disruption, increased vascular permeability, pulmonary edema, and impaired gas exchange, thereby aggravating sepsis‐induced lung injury.

As summarized in Figure 3, sepsis‐related MAM disruption promotes Ca^2+^ dysregulation, ROS accumulation, and mitochondrial DNA (mtDNA) release. These signals converge on NLRP3 inflammasome activation and drive IL‐1β/IL‐18–dominated cytokine storm, thereby exacerbating pulmonary barrier injury and gas‐exchange impairment.

#### 3.4.2. Immunological Signaling Roles of mtDNA and ROS

Dysfunction of mitochondria‐associated endoplasmic reticulum membranes can lead to the release of mtDNA into the cytoplasm, where it acts as a damage‐associated molecular pattern (DAMP) to trigger immune activation. When MAM function is compromised, mitochondrial homeostasis is disrupted, potentially resulting in mitochondrial membrane rupture and the subsequent release of mtDNA. This cytosolic mtDNA can be recognized by intracellular DNA sensors such as cyclic GMP–AMP synthase (cGAS), which in turn activates the stimulator of interferon genes (STING) signaling pathway. Activation of this pathway promotes the production of interferons and inflammatory cytokines, thereby eliciting a robust innate immune response [44].

As a DAMP, mtDNA also contributes to inflammation by activating the NLRP3 inflammasome and the NF‐κB signaling pathway, leading to the enhanced secretion of IL‐1β and amplification of the inflammatory cascade [45]. In parallel, excessive generation of ROS serves as an important signaling mechanism that modulates immune cell activation and cytokine expression. ROS are natural byproducts of cellular metabolism and, at physiological levels, are essential for intracellular signaling. However, excessive ROS accumulation results in oxidative damage and cell death. Elevated ROS can promote the activation and function of immune cells, including T cells and macrophages, through multiple mechanisms [46].

In pathological conditions such as sepsis‐induced lung injury, ROS production is closely associated with immune cell activation. ROS can oxidatively modify proteins and lipids, altering their structure and function, which in turn affects immune signaling and cell activation states [47]. Furthermore, ROS enhance cytokine expression, thereby intensifying the inflammatory response and contributing to the progression of tissue injury [48].

#### 3.4.3. Regulation of Immune Cell Function by MAMs

Mitochondria‐associated endoplasmic reticulum membranes play a pivotal role in the polarization of macrophages, and dysfunction of MAMs has been shown to promote polarization toward the proinflammatory M1 phenotype, thereby amplifying inflammatory responses—a phenomenon documented in various inflammatory diseases [12, 49]. The activation state of macrophages is closely linked to the structural and functional integrity of MAMs. Abnormalities in MAMs can lead to dysregulated calcium signaling, which in turn alters macrophage activation and impairs their capacity to secrete inflammatory cytokines. Additionally, MAMs influence macrophage function through the regulation of cellular energy metabolism. The functional state of MAMs is tightly associated with macrophage metabolic reprogramming; when MAM integrity is compromised, macrophage metabolism shifts toward a more proinflammatory phenotype—a hallmark feature observed in sepsis [50, 51].

Neutrophils are likewise regulated by MAMs. By modulating energy metabolism and calcium signaling, MAMs influence neutrophil chemotaxis and bactericidal activity. In sepsis, neutrophil activation is frequently accompanied by alterations in MAM function, which can diminish the efficiency of pathogen clearance. Dysfunctional MAMs may result in either excessive or suppressed neutrophil activation, thereby exacerbating inflammatory responses and worsening the prognosis of sepsis [52, 53].

Through the regulation of calcium release, MAMs enhance the activity of macrophages and neutrophils, which is essential for effective pathogen elimination and the modulation of inflammatory responses [54, 55]. Furthermore, MAMs play a crucial role in the metabolic reprogramming of macrophages and other immune cells, influencing cellular energy supply and metabolic state. In the septic microenvironment, significant alterations in cellular energy metabolism directly impact immune cell activation and apoptosis [20, 56]. MAM dysfunction can lead to excessive apoptosis of immune cells, thereby weakening the host immune response. This phenomenon is particularly evident in severe infections such as sepsis, where MAM dysregulation exacerbates immune cell injury and apoptosis, ultimately influencing disease progression and clinical outcomes [53, 57].

#### 3.4.4. MAMs and Immune Cell Fate Determination: From Organelle Signaling to Immune Phenotypes

Mitochondria‐associated endoplasmic reticulum membranes serve not only as critical platforms for intracellular signal integration but also play pivotal roles in immune cell fate determination by regulating calcium (Ca^2+^) transfer, mitochondrial metabolism, and oxidative stress responses. Accumulating evidence suggests that the structural and functional status of MAMs can directly influence metabolic reprogramming and inflammatory phenotypes of immune cells, thereby profoundly contributing to immune dysregulation and lung injury during sepsis. Figure 2 illustrates the proposed mechanisms by which MAM dysfunction links organelle‐derived signals to immune cell phenotypic alterations in sepsis.

##### 3.4.4.1. MAMs and Macrophage Polarization

Macrophage polarization is tightly coupled to cellular metabolic states, and MAMs play a central regulatory role in this process by modulating ER–mitochondria Ca^2+^ signaling and mitochondrial oxidative phosphorylation [58]. Under homeostatic conditions, intact MAMs facilitate controlled Ca^2+^ transfer to mitochondria, thereby sustaining tricarboxylic acid cycle activity and oxidative phosphorylation efficiency, which favors the development of anti‐inflammatory and tissue‐repair–associated M2‐like phenotypes. In contrast, during sepsis, disruption of MAM structure leads to aberrant Ca^2+^ transfer and mitochondrial dysfunction, driving macrophage metabolism toward a glycolysis‐dependent state accompanied by excessive ROS production [59]. This metabolic reprogramming activates inflammatory transcriptional pathways, including NF‐κB and HIF‐1α, thereby promoting polarization toward the proinflammatory M1 phenotype and enhancing the production of cytokines such as TNF‐α, IL‐6, and IL‐1β. Consequently, these changes exacerbate pulmonary inflammation and tissue injury.

##### 3.4.4.2. MAMs and Neutrophil Activation and Neutrophil Extracellular Trap (NET) Formation

Neutrophil chemotaxis, degranulation, and antimicrobial functions are highly dependent on dynamic Ca^2+^ signaling and oxidative burst responses [60]. By regulating Ca^2+^ microdomain signaling and mitochondrial ROS generation, MAMs actively participate in neutrophil activation. Under septic conditions, MAM dysfunction can result in amplified or dysregulated Ca^2+^ signaling accompanied by excessive mitochondrial ROS production, thereby promoting aberrant neutrophil activation. In particular, Ca^2+^ overload and ROS accumulation are recognized as key drivers of NET formation [61]. MAM‐associated mitochondrial dysfunction may therefore enhance NET formation, leading to aggravated pulmonary microvascular injury, increased vascular permeability, and inflammatory cell infiltration, ultimately worsening sepsis‐induced lung injury.

##### 3.4.4.3. MAMs and T Cell Functional Exhaustion

T cell activation and effector functions require sustained and precisely regulated Ca^2+^ signaling as well as adequate mitochondrial energy supply [62]. By maintaining Ca^2+^ signaling stability and mitochondrial metabolic integrity, MAMs support T cell proliferation and cytokine production. In the context of sepsis, disruption of MAM structure and function leads to mitochondrial Ca^2+^ imbalance and impaired oxidative phosphorylation, resulting in energy deficiency and persistent metabolic stress. This metabolic dysregulation compromises T cell effector functions and promotes the development of functional exhaustion or apoptosis. Moreover, the release of mtDNA and the ROS‐enriched inflammatory microenvironment associated with MAM dysfunction may further exacerbate T cell exhaustion, thereby contributing to the immunosuppressive state observed in the later stages of sepsis [63].

Collectively, MAMs act as central regulatory hubs that integrate Ca^2+^ signaling, mitochondrial metabolism, and oxidative stress responses to govern macrophage polarization, neutrophil activation, and T cell functional maintenance. Dysregulation of MAM function drives aberrant immune cell phenotypes, amplifies inflammatory responses, and impairs effective host defense, representing a critical organelle‐based mechanism underlying sepsis‐associated lung injury and immune dysfunction.

### 3.5. Advances in Targeting MAMs as Therapeutic Strategies for Sepsis‐Induced Lung Injury

#### 3.5.1. Strategies for Targeting MAM Structural Restoration

In sepsis‐induced lung injury, increasing attention has been paid to therapeutic strategies aimed at restoring the structural integrity of mitochondria‐associated endoplasmic reticulum membranes. The stability of MAM architecture depends on the coordinated expression and function of key tethering proteins, among which MFN2 plays a central role in maintaining ER–mitochondria contacts and regulating Ca^2+^ transfer and mitochondrial homeostasis. Previous studies have shown that downregulation of MFN2 is closely associated with MAM disruption, leading to reduced ER–mitochondria coupling and impaired Ca^2+^ signaling efficiency, thereby exacerbating cellular dysfunction and inflammatory responses in sepsis [64].

Tetramethylpyrazine (TMP) has been reported to promote MFN2 transcription and inhibit its degradation, resulting in restoration of MAM structural stability, improvement of mitochondrial function, and attenuation of sepsis‐associated lung injury [8]. In addition, pharmacological and gene‐based approaches targeting MAM‐related proteins have been proposed as potential strategies to enhance ER–mitochondria coupling and alleviate endothelial inflammation. For example, activation of the sigma‐1 receptor (Sig1R) has been shown to improve MAM function and mitigate pathological features of sepsis‐induced lung injury [9].

#### 3.5.2. Modulating MAM Function to Improve Mitochondrial Metabolism

In addition to direct restoration of MAM structure, modulation of MAM‐associated functional disturbances represents another important therapeutic avenue. Oxidative stress is a key contributor to MAM dysfunction and mitochondrial metabolic impairment in sepsis. Antioxidants can alleviate mitochondrial membrane damage by scavenging excessive ROS, thereby improving mitochondrial bioenergetics and cellular survival. Urolithin A (UA) has been shown to regulate MAM‐associated Ca^2+^ homeostasis, reduce ER stress, and enhance cellular resistance to metabolic and inflammatory insults [65]. In other disease models, antioxidant treatment has also been reported to increase mitochondrial Ca^2+^ uptake capacity and improve mitochondrial metabolic function [66].

Ca^2+^ dysregulation is a hallmark of MAM functional impairment. Within MAMs, IP3Rs and VDACs coordinate ER‐to‐mitochondria Ca^2+^ transfer, and their dysfunction can lead to mitochondrial Ca^2+^ overload, metabolic collapse, and apoptosis [67]. Accordingly, targeting Ca^2+^ transport pathways at MAMs is considered a promising strategy for improving mitochondrial metabolism. Moreover, antioxidant interventions have been shown to restore MAM function, reduce mitochondrial Ca^2+^ overload and ROS generation, and consequently attenuate apoptosis and tissue injury [59].

#### 3.5.3. Regulation of MAM Dynamics and Stress Adaptation

It is increasingly recognized that MAM abundance and function must be finely balanced to maintain cellular homeostasis. Excessive or aberrant MAM formation may exacerbate ER stress and cellular injury under pathological conditions. Dendrobine has been shown to exert protective effects in models of sepsis‐induced acute lung injury (SI‐ALI) by regulating family with sequence similarity 134 member B (FAM134B)–mediated ER‐phagy, thereby suppressing excessive MAM formation and alleviating ER stress [10]. These findings suggest that modulation of MAM dynamics and stress adaptation processes may represent a viable therapeutic strategy for sepsis‐associated lung injury.

#### 3.5.4. Future Perspectives and Translational Challenges

Despite promising evidence from experimental studies, several challenges remain in translating MAM‐targeted therapeutic strategies into clinical practice. First, MAM functions exhibit marked cell‐type specificity, varying among alveolar epithelial cells, endothelial cells, and immune cells, and their roles may differ across distinct stages of sepsis. Second, most current interventions rely on indirect modulation of MAM function, while highly specific, safe, and controllable MAM‐targeted agents remain limited. In addition, standardized and reliable methods for assessing MAM integrity and function in clinical samples are still lacking, which hamper patient stratification and therapeutic monitoring [57].

## 4. Conclusion

The ER–mitochondria contact sites (MAMs) play a vital role in regulating intracellular calcium homeostasis, energy metabolism, and immune signaling and are key participants in pathological processes such as sepsis‐induced lung injury. Sepsis leads to structural and functional abnormalities of MAMs, resulting in calcium signaling dysregulation, mitochondrial damage, and exacerbated inflammation, thereby intensifying pulmonary injury. Moreover, MAMs are involved in critical signaling pathways including inflammasome activation, ROS production, and mtDNA release, positioning them as central regulators of immune responses.

Despite recent advances in understanding MAM biology, discrepancies among studies under varying experimental conditions remain. Future investigations should therefore integrate multiple factors to elucidate the precise molecular mechanisms governing MAM function in sepsis. Therapeutic strategies targeting MAMs have shown promising potential; however, translating these findings from basic research to clinical application will require sustained effort and interdisciplinary collaboration.

Overall, research on MAMs provides novel insights into the pathogenesis and treatment of sepsis‐induced lung injury, offering new therapeutic avenues that may improve patient outcomes and advance the development of clinical medicine.

**Nomenclature**
ATP: Adenosine triphosphate
DAMP: Damage‐associated molecular pattern
ER: Endoplasmic reticulum
FAM134B: Family with sequence similarity 134 member B
GRP75: Glucose‐regulated protein 75
IL: Interleukin
IP3R: Inositol 1,4,5‐trisphosphate receptor
M1: M1 macrophage phenotype
MAMs: Mitochondria‐associated membranes
MFN2: Mitofusin‐2
mtDNA: Mitochondrial DNA
NF‐κB: Nuclear factor kappa‐B
NLRP3: NLR family pyrin domain containing 3
ROS: Reactive oxygen species
Sig1R: Sigma‐1 receptor
STING: Stimulator of interferon genes
TMP: Tetramethylpyrazine
UA: Urolithin A
VDAC: Voltage‐dependent anion channel
PKC: Protein kinase C
PLA_2_: Phospholipase A_2_
SIRS: Systemic inflammatory response syndrome.

## Author Contributions

**Yihao Wang:** conceptualization of the review topic, literature search and collection, data extraction, original draft writing. **Jingran Yang:** literature screening, formal analysis of the studies, figure preparation, writing assistance for the manuscript. **Xia Li**: supervision, critical revision of the manuscript for important intellectual content, project administration, funding acquisition.

## Funding

This research topic has been sponsored by the 2024 Nantong University Clinical Medicine Special Research Fund Project (Grant 2024JY015) and the 2024 Medical Research Grant of the Yancheng Municipal Health Commission (Grant YK2024051).

## Disclosure

All authors have read and approved the final version of the manuscript and agree to be accountable for all aspects of the work, ensuring that questions related to the accuracy or integrity of any part of the work are appropriately investigated and resolved.

## Consent

The authors have nothing to report.

## Conflicts of Interest

The authors declare no conflicts of interest.

## Data Availability

Data sharing is not applicable to this article as no data were created or analyzed in this study.
